# Poly-γ-Glutamic Acid from a Novel *Bacillus subtilis* Strain: Strengthening the Skin Barrier and Improving Moisture Retention in Keratinocytes and a Reconstructed Skin Model

**DOI:** 10.3390/ijms26030983

**Published:** 2025-01-24

**Authors:** Hyun-Ju Ko, SeoA Park, Eunjin Shin, Jinhwa Kim, Geun Soo Lee, Ye-Jin Lee, Sung Min Park, Jungno Lee, Chang-Gu Hyun

**Affiliations:** 1 Bio Convergence R&D Center, CoSeedBioPharm Corporation, Heungdeok-gu, Cheongju 28161, Chungbuk, Republic of Korea; ok6336@hanmail.net (H.-J.K.); tnqls6420@daum.net (S.P.); simple3507@naver.com (E.S.); smp@coseed.co.kr (S.M.P.); 2 R&D Center, ItsHanbul, 62, 547, Daeseong-ro, Samseong-myeon, Eumseong-gun 27651, Chungbuk, Republic of Korea; kjhi@itshanbul.com (J.K.); aria@itshanbul.com (G.S.L.); 3 Jeju Inside Agency and Cosmetic Science Center, Department of Chemistry and Cosmetics, Jeju National University, Jeju 63243, Jeju-do, Republic of Korea; zhftmak615@naver.com

**Keywords:** *Bacillus subtilis*, Gotjawal wetland, poly-γ-glutamic acid, skin barrier, reconstructed skin model

## Abstract

A novel *Bacillus subtilis* HB-31 strain was isolated from Gotjawal Wetland in Jeju Island, Republic of Korea. A mucus substance produced by this strain was identified as high-molecular-weight poly-γ-glutamic acid (γ-PGA) using NMR, Fourier transform infrared spectroscopy, and size-exclusion chromatography/multi-angle light scattering analyses. We evaluated whether γ-PGA strengthened the skin barrier using keratinocytes and a reconstructed skin model. In keratinocytes, γ-PGA treatment dose-dependently increased the mRNA expression of skin barrier markers, including filaggrin, involucrin, loricrin, serine palmitoyl transferase, fatty acid synthase, and 3-hydroxy-3-methylglutaryl coenzyme A reductase. γ-PGA also enhanced hyaluronic acid synthesis by upregulating hyaluronic acid synthase-1, -2, and -3 mRNA levels and promoted aquaporin 3 expression, which is involved in skin hydration. In the reconstructed skin model, topical application of 1% γ-PGA elevated filaggrin, involucrin, CD44, and aquaporin 3 expression, compared to the control. These results suggest that the newly isolated HB-31 can be used as a commercial production system of high-molecular-weight γ-PGA, which can serve as an effective ingredient for strengthening the skin barrier and improving moisture retention. Further research is needed to explore the long-term effects of γ-PGA on skin health and its application in treating skin conditions.

## 1. Introduction

In light of the severity of environmental issues and the growing number of environmental regulations, the potential of eco-friendly biodegradable macromolecules has been actively explored. Biodegradable macromolecules that have received considerable attention include substances produced by microbes, including the polyester polyhydroxyalkanoate, polysaccharides such as pullulan and microbial cellulose, and the polypeptide poly-γ-glutamic acid (γ-PGA) [1]. Of these macromolecules, γ-PGA is widely recognized as a mucous substance in the traditional Japanese food natto and the Korean food cheonggukjang. γ-PGA is a large polymer in which the γ-carboxylic acid and α-amino groups of glutamic acid are linked via amide linkages. Its molecular weight is around 100–1000 kDa, depending on the microbial species of the source [2]. As a fermentation product of several Bacillus spp., including *B. subtilis*, γ-PGA is secreted extracellularly during incubation; it is non-toxic to humans and the environment and is water-soluble and biodegradable [2].

In recent years, γ-PGA has been successfully utilized in the beauty and wellness sectors. Therefore, low-cost, high-efficiency γ-PGA production has garnered considerable attention. Microbial synthesis of γ-PGA provides advantages over traditional chemical methods, including better control over polymer molecular weight and stereochemistry [3]. It is also performed under mild conditions, reducing energy costs and environmental impact compared to chemical synthesis [3]. γ-PGA has several beneficial effects on the skin. When applied topically, it creates a moisture-retaining layer that effectively prevents water loss [4]. Additionally, its strong antibacterial properties contribute to maintaining a healthy skin pH value [5]. γ-PGA also inhibits the activity of tyrosinase, an enzyme responsible for skin melanin production [6]. Despite its application in the cosmetic industry, the effect of high-molecular-weight γ-PGA on the protective barrier of the skin remains largely unexplored. Consequently, there is great value in discovering new γ-PGA-producing organisms from natural sources, verifying the structure and molecular mass of γ-PGA that they produce, and exploring its potential uses.

With increasing interest in the function and importance of the skin barrier, research on the development of skin cosmetic ingredients, which has previously focused on antioxidative mechanisms, has gradually diversified to include the recovery and regulation of the damaged skin barrier. Researchers are now investigating compounds that can stimulate the production of key barrier components, such as ceramides and lipids, which play crucial roles in maintaining skin hydration and protecting against external stressors [7,8,9].

The skin serves as the body’s primary protective barrier, conserving water and shielding against external factors. The outermost epidermal layer, the stratum corneum (SC), guards against dry environments; it consists of structurally important corneocytes and a lipid matrix filling the spaces between them [10]. SC lipids, crucial for barrier function, include ceramides and cholesterol, which influence SC integrity, and free fatty acids, which considerably affect bilayer formation and pH [10].

During differentiation, keratinocytes become flat anucleated corneocytes. Concurrently, differentiation-promoting factors such as involucrin in the granular and upper spinous layers [11] and loricrin and filaggrin in the granular and horny layers [12] are expressed. This process leads to the aggregation of keratin filaments into a flat cornified cell envelope, forming a strong physical and permeable skin barrier [13]. Filaggrin degrades into natural moisturizing factors, such as pyrrole carboxylic acid and trans-urocanic acid, which aid in skin hydration and pH neutralization, and exerts anti-inflammatory effects in the horny layer [14,15].

In addition to the natural moisturizing factors derived from filaggrin, the skin acts as a moisturizing barrier through various moisturizing factors such as hyaluronic acid (HA), urea, citrate, lactate, and glycerol [16]. HA is a high-molecular-weight compound of 200,000–400,000 Da, which not only prevents moisture evaporation from the epidermis and maintains skin elasticity but also plays a role in cell movement and nutrient storage and diffusion [17].

Aquaporin 3 (AQP3), a protein responsible for skin moisture, elasticity, and skin regeneration, is present in the epidermis and supplies moisture to cells [18]. Various skin conditions, such as atopic dermatitis [19], psoriasis [20], vitiligo [21], and chronic skin pruritus [22], are related to the expression of AQP3 in keratinocytes. As skin ages, the decreased expression of AQP3 in the epidermis reduces skin hydration [18,23]. Increased expression of AQP3 is reported to prevent and alleviate skin conditions [24].

The effectiveness of a substance on the skin can be assessed using different biological methods [25]. Although monoculture is easy to use, it is insufficient for studying the physiological structure of the skin. The use of 3D models that simulate skin tissue with a fully differentiated epidermis and fibroblast-populated dermal equivalent can overcome this issue to some extent. Numerous studies involving the use of these skin models have demonstrated that these models reproduce the biological events that occur when the skin is exposed to substances in a manner similar to that found in in vivo situations [26,27,28,29,30]. These skin models have been shown to be useful tools for the evaluation of products that can be applied topically [31].

The objective of this study was to identify a novel γ-PGA-producing organism from the natural environment and examine its effects on skin barrier function and hydration. This investigation emphasizes the potential of γ-PGA as an environmentally friendly and sustainable ingredient for cosmetic applications.

## 2. Results and Discussion

### 2.1. Isolation and Identification

We collected bacteria from Gotjawal Wetland, Jeju Island, Republic of Korea, to isolate native fermenting bacteria with diverse functional and physiological activities. We purified approximately 15 species through heat treatment and then selected and identified the strain with the highest viscosity in tryptic soy agar (TSA) medium. Using 16S rRNA gene sequencing and the basic local alignment search tool (BLAST), we identified the selected strain as *B. subtilis*. However, *Bacillus* spp. can produce spores and survive in diverse environments; hence, species diversity can be markedly high depending on the environment. Even with 100% homology in 16S rRNA sequencing, different *Bacillus* spp. can be confused with one another [32]. Accordingly, we analyzed sugar metabolism using an API 50CH kit, which also revealed the strain as *B. subtilis*, consistent with the 16S rRNA sequencing results (Sequencing analysis report held). Therefore, this strain was named *B. subtilis* HB-31. The final selected strain was registered at the Korean Collection for Type Cultures of the Korea Research Institute of Bioscience and Biotechnology as *B. subtilis* KCTC 13486BP.

### 2.2. Identification of γ-PGA

The isolated compound was a white powder with a ^1^H-, ^13^C-NMR spectrum showing alpha α-CH [δ_H_ 3.288 (m), δ_C_ 53.89], β-CH_2_ [δ_H_ 2.26 (t, *J* = 6.0, 6.8 Hz), δ_C_ 25.51], γ- CH_2_ [δ_H_ 1.854 (t), *J* = 6.0, 6.8 Hz, δ_C_ 30.04], NH [δ_C_ 173.90], and COOH [δ_C_ 177.12].

**Figure 1:**
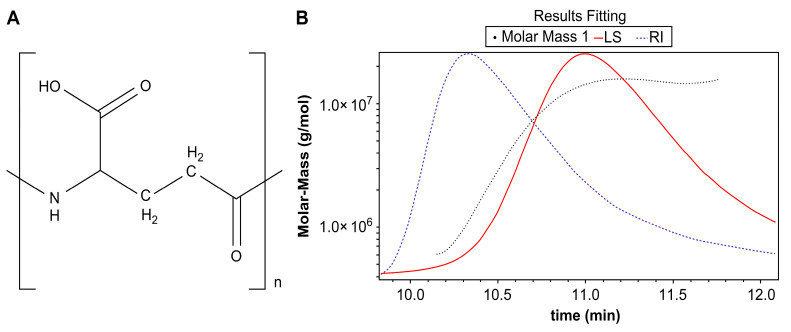
(**A**) Structure of γ-PGA and (**B**) absolute molecular weight measurements using a multi-angle light scattering detector and a refractive index (RI) detector.

Fourier transform infrared spectroscopy (FT-IR) aids in the identification of the functional groups of organic and non-organic compounds. The FT-IR data showed peaks at 3200–3500 (–OH, broad), 3209 (–NH, broad), 2900 (CH_2_, single), 1637 (C=O, single), and 1504 cm^−1^ (–NH, single). The structure of the compound was that of γ-PGA, as reported in the literature (Figure 1A) [33].

The absolute molecular weight of the isolated compound was measured using size exclusion chromatography (SEC)/multi-angle light scattering (MALS). The retention time was detected as a single peak with the MALS detector at 10.138–11.772 min and the absolute molecular weight was determined to be 6.975 × 10^6^ (±9.643) Da (6975 kDa) (Figure 1B). This sample is considered to be the largest polymer form of γ-PGA reported to date.

The high molecular weight of this γ-PGA suggests exceptional polymerization efficiency during biosynthesis. This large polymer size may contribute to unique physicochemical properties and potential applications in various fields, such as biomedicine, the food industry, and environmental remediation. Further studies are warranted to explore the relationship between the molecular weight and functional characteristics of this γ-PGA sample.

### 2.3. Cytotoxicity of γ-PGA in Keratinocytes

**Figure 2:**
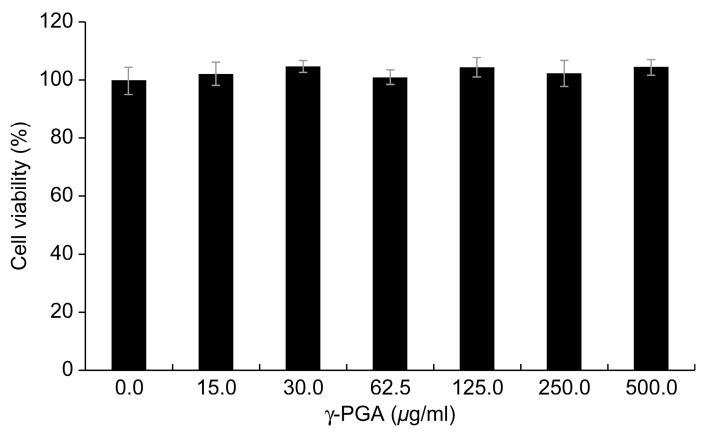
Cytotoxicity of γ-PGA in keratinocytes. Data are presented as the mean ± SD; n = 3.

γ-PGA did not induce evident cytotoxicity in the keratinocytes until a concentration of 500 µg/mL (Figure 2). Therefore, we conducted all subsequent experiments in keratinocytes with γ-PGA at this concentration, which did not induce toxicity in any of the treatment groups.

### 2.4. Effect of γ-PGA on Physical Skin Barrier-Related Markers in Keratinocytes

The keratinocyte differentiation markers filaggrin, involucrin, and loricrin play important roles in the formation and functional maintenance of the cornified cell envelope, which is the physical barrier of the skin. Reduced expression of these factors has been associated with skin conditions such as atopic dermatitis and psoriasis, owing to impaired skin barrier function.

**Figure 3:**
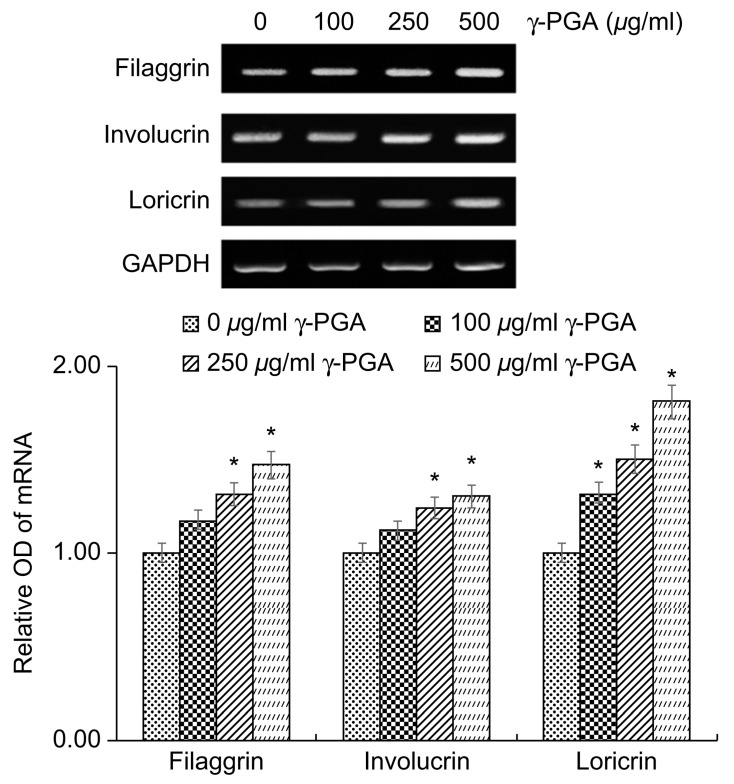
Effect of γ-PGA on filaggrin, involucrin, and loricrin mRNA expression in keratinocytes. mRNA expression was determined using reverse transcription polymerase chain reaction, with *GAPDH* mRNA as an internal control. Data are presented as the mean ± SD; n = 3; * *p* < 0.05 vs. untreated control.

To determine the effects of γ-PGA on physical skin barrier function, we treated the keratinocytes with γ-PGA and incubated them for 24 h; we then performed reverse transcription polymerase chain reaction (RT-PCR) analysis to measure the expression levels of these differentiation markers. Gene expression levels were quantified based on the expression levels in the control group relative to the level of the housekeeping gene *GAPDH*. γ-PGA-treated keratinocytes exhibited higher expression of filaggrin, involucrin, and loricrin mRNA than the untreated control group in a dose-dependent manner (* *p* < 0.05; Figure 3). These results indicate that γ-PGA could improve and strengthen the physical barrier of the skin by promoting keratinocyte differentiation.

### 2.5. Effect of γ-PGA on Permeability Skin Barrier-Related Markers in Keratinocytes

The lipids of the SC are different from those of normal biological membranes in that they do not contain phospholipids, and they are mainly composed of ceramides, cholesterol, and free fatty acids. Normal biological membranes that contain phospholipids, sphingomyelin, and cholesterol do not function as barriers because small substances that are soluble in water can easily pass through them. However, the SC, which does not contain phospholipids, possesses lipids that are well connected in a straight line, and therefore it acts as an excellent barrier that prevents the penetration of water-soluble or small substances. However, in individuals with chronic inflammatory conditions, such as atopic dermatitis, and those with dry or aged skin, the lipid level of the SC decreases and the skin barrier function weakens.

The intercellular lipid components of the barrier are newly synthesized during keratinocyte differentiation. Among the epidermal lipids, cholesterol synthesis is associated with increased 3-hydroxy-3-methylglutaryl coenzyme A (*HMG-CoA*) reductase activity. Increased epidermal fatty acid synthesis is associated with increased activity of acetyl-CoA carboxylase and fatty acid synthase (*FAS*), and increased epidermal ceramide synthesis is associated with serine palmitoyl transferase (*SPT*) activity [34].

**Figure 4:**
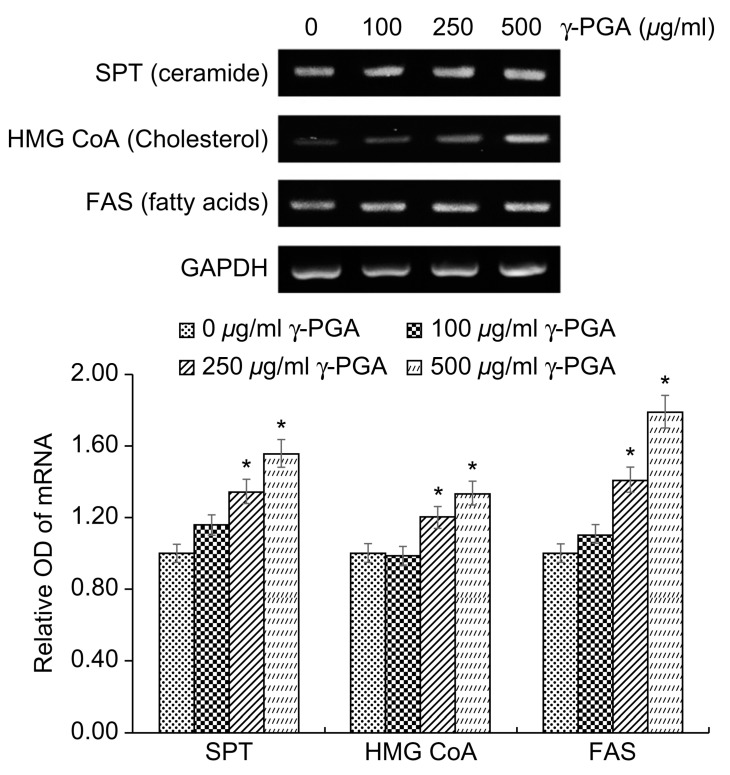
Effect of γ-PGA on *SPT, FAS*, and *HMG-CoA* mRNA in keratinocytes. mRNA expression was determined using RT-PCR, with GAPDH mRNA as an internal control. Data are presented as the mean ± SD; n = 3; * *p* < 0.05 vs. untreated control.

To determine the effects of γ-PGA on epidermal lipid synthesis, we performed RT-PCR analysis to measure the expression levels of *SPT*, *FAS*, and *HMG-CoA* mRNA after treatment of keratinocytes with γ-PGA and incubation for 24 h. Gene expression levels were quantified based on the expression levels in the control group relative to the level of the housekeeping gene *GAPDH*. γ-PGA-treated keratinocytes exhibited higher expression of *SPT*, *FAS*, and *HMG-CoA* mRNA than the untreated control group in a dose-dependent manner (* *p* < 0.05; Figure 4). These results indicate that γ-PGA can strengthen the epidermal permeability barrier by increasing the synthesis of epidermal lipids.

### 2.6. Effect of γ-PGA on Hyaluronic Acid Synthesis in Keratinocytes

HA is mainly synthesized by hyaluronic acid synthase (HAS) in keratinocytes and fibroblasts and accumulates in the extracellular matrix [35]. To date, the known HAS genes have been reported to comprise three types, referred to as *HAS-1*, *HAS-2*, and *HAS-3* [36]. Defects in the moisture barrier due to the decreased expression of these genes have been reported to cause skin aging, such as epidermal atrophy, wrinkle formation, decreased skin moisture, and decreased elasticity [36].

**Figure 5:**
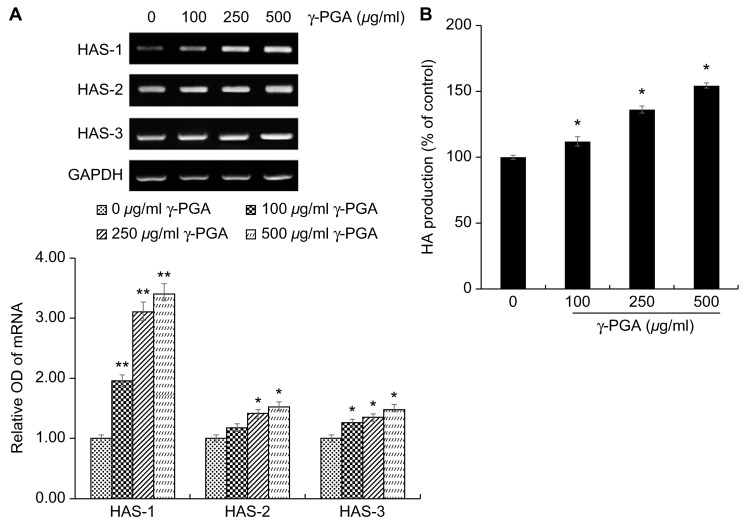
Effect of γ-PGA on HA synthesis in keratinocytes. (**A**) mRNA expression was determined using RT-PCR, with *GAPDH* mRNA used as the internal control. (**B**) HA secretion level by keratinocytes was determined using ELISA kit (CUSABIO, Wuhan, China). Data are presented as the mean ± S.D.; n = 3; * *p* < 0.05 vs. untreated control. ** *p* < 0.01 vs. untreated control.

After treating keratinocytes with γ-PGA, to determine the effects of γ-PGA on HA synthesis, we performed RT-PCR. Gene expression levels were quantified based on the expression levels in the control group relative to the level of the housekeeping gene *GAPDH*. γ-PGA-treated keratinocytes exhibited higher expression of *HAS-1*, *HAS-2*, and *HAS-3* mRNA than the untreated control group in a dose-dependent manner (* *p* < 0.05, ** *p* < 0.01; Figure 5A). HA synthesis, which was promoted, was quantified using an enzyme-linked immunosorbent assay kit. The γ-PGA-treated group showed a significant increase in HA production compared to the untreated group at different treatment concentrations (* *p* < 0.05; Figure 5B). These results indicate that γ-PGA can strengthen the moisture barrier by increasing the synthesis of HA.

### 2.7. Effect of γ-PGA on AQP3 Expression in Keratinocytes

AQP3 plays an important role in supplying moisture to the skin, making it a new target for skin moisturizers [37]. As skin ages, AQP3 expression decreases in normal human epidermal keratinocytes [18]. Therefore, natural active substances that increase AQP3 expression may be effective moisturizers in anti-aging cosmetics [24].

To confirm whether the expression of AQP3 increased, fluorescent immunocytochemistry analysis with an anti-AQP3 antibody was performed, and the results were visualized with a fluorescence microscope to obtain qualitative data. There was an increase in the expression of AQP3 in keratinocytes upon exposure to γ-PGA at different treatment concentrations for 24 h. In addition, after treating the keratinocytes with γ-PGA, we performed RT-PCR to determine the effects of γ-PGA on *AQP3* mRNA expression.

**Figure 6:**
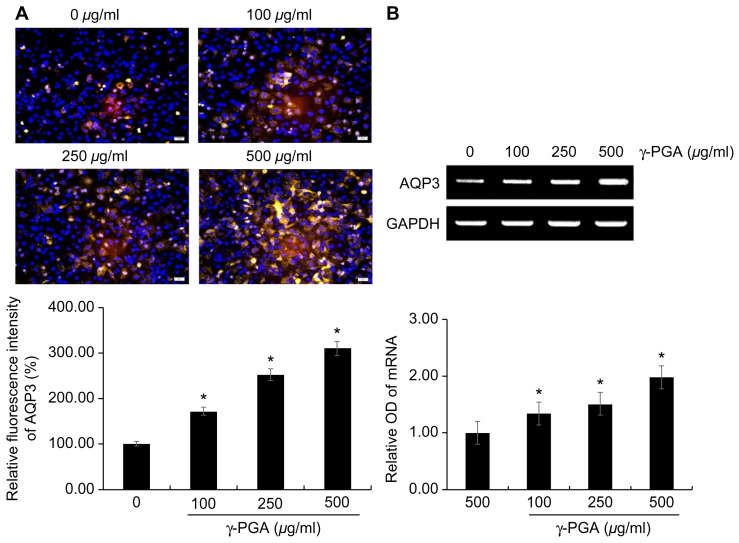
Effect of γ-PGA on AQP3 expression in keratinocytes. (**A**) AQP3 protein expression observed using a THUNDER Imager Tissue microscope. Yellow fluorescence indicates the AQP3 protein and blue fluorescence indicates DAPI staining sites (nuclear regions). (**B**) mRNA expression was determined using RT-PCR, with *GAPDH* mRNA used as the internal control. Data are presented as the mean ± SD; n = 3; * *p* < 0.05 vs. untreated control. Scale bar: 50 µm.

The expression of AQP3 protein (Figure 6A) and mRNA (Figure 6B) qualitatively increased in a γ-PGA concentration-dependent manner compared with that in the untreated control group (* *p* < 0.05; Figure 6A,B).

### 2.8. Effect of γ-PGA on Skin Barrier-Related Markers in a Reconstructed Skin Model

**Figure 7:**
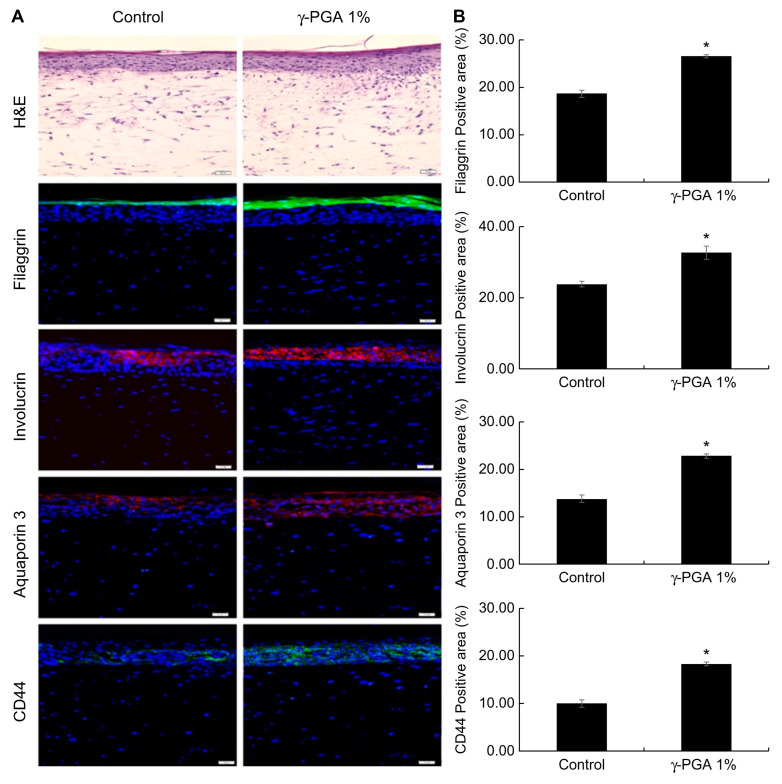
Effect of γ-PGA on skin barrier and moisture level in the reconstructed skin. (**A**) Filaggrin, involucrin, aquaporin, and CD44 protein expression observed using the THUNDER Imager Tissue microscope. The color fluorescence indicates filaggrin (green), involucrin (red), aquaporin (red), and CD44 (green), while the blue fluorescence indicates DAPI staining sites (nuclear regions). (**B**) Calculation graph. Data are presented as the mean ± SD; n = 3; * *p* < 0.05 vs. untreated control. Scale bar: 50 µm.

Reconstructed skin has a structure similar to that of human skin, and keratinocytes cultured on the dermis form a horny layer through proliferation and differentiation. Using these characteristics, we examined the effectiveness of γ-PGA in strengthening the skin barrier. Filaggrin and involucrin play important roles as structural proteins as part of the skin barrier. Reduced expression of these proteins damages the skin barrier. Based on our immunofluorescent staining results, reconstructed skin treated with 1% γ-PGA exhibited elevated expression of filaggrin and involucrin compared to control-treated skin (* *p* < 0.05; Figure 7). In addition, the horny layer thickness increased in γ-PGA-treated skin compared with that in control-treated skin, suggesting that the skin barrier function was enhanced.

CD44, a transmembrane glycoprotein present in the cell membrane, is a receptor for HA. It has excellent water-retention capacity and is involved in cell proliferation and migration [38]. It has been reported that the expression locations of CD44 and HA in the human skin are similar and that as the amount of HA increases, the amount of CD44 also increases [39]. CD44 is expressed in the basal layer through granules. To confirm that CD44 expression is increased by γ-PGA throughout the epidermis, an immunohistofluorescence analysis was conducted using an antibody that detects CD44. Reconstructed skin treated with 1% γ-PGA exhibited elevated expression of CD44 compared to the control-treated skin (* *p* < 0.05; Figure 7).

In the epidermis, AQP3 is expressed from the basal layer to the granular layer but disappears in the SC. The distribution of AQP3 expression correlates with water level; the basal and suprabasal living layers contain 75% water, whereas the SC contains only 10–15% water. Decreased expression of AQP3 in the skin leads to dry skin, decreased skin elasticity, and delayed barrier recovery [40].

In order to examine the effect of γ-PGA on AQP3 expression, an immunohistofluorescence analysis was conducted on the reconstructed skin section. Based on immunohistofluorescence staining results, the reconstructed skin treated with 1% γ-PGA exhibited elevated expression of AQP3 compared to the control-treated skin (* *p* < 0.05; Figure 7).

γ-PGA can be an effective treatment option for skin that has a weak barrier or is dehydrated by increasing the expression of FLG, IVL, CD44, and AQP3.

The novelty of this study lies in the characterization of γ-PGA produced by the newly isolated *Bacillus* sp. from Gotjawal Wetland, Jeju Island, rather than the existing *Bacillus* spp. derived from cheonggukjang or natto, and the confirmation of the effect of the substance on the skin barrier and moisture level in a reconstructed skin model. The discovery of this new γ-PGA-producing organism opens up exciting possibilities for sustainable and eco-friendly production methods. The γ-PGA produced by the bacterial strain from Gotjawal Wetland in Jeju shows potential as a cosmeceutical ingredient for improving skin barrier function and hydration; despite this, additional research is needed to fully understand its properties and advantages over other γ-PGA sources in skincare. It is also necessary to characterize this organism and optimize γ-PGA production processes for commercial use in cosmetics. Since the high-molecular-weight γ-PGA secreted by this strain differs from previously reported γ-PGA, its effects on skin may vary. Future research should include comparative studies to explore the relationship between molecular weight and dermatological efficacy.

A significant limitation of this study is the absence of in vivo data to support and validate the in vitro findings. Although in vitro experiments offer valuable insights into cellular processes and their potential impacts, they may not fully reflect the intricacies of living organisms. The skin, a complex organ with multiple layers and interactions with various bodily systems, might react differently to γ-PGA in living organisms than in isolated cell cultures. This limitation highlights the need for thorough in vivo research to verify the effects of γ-PGA on skin health.

To address this limitation, the authors of future studies should focus on conducting well-designed clinical trials involving human subjects. Such studies should evaluate the influence of γ-PGA on diverse cutaneous parameters, considering variables such as skin phenotypes, age demographics, and environmental factors. Through the integration of in vitro, in vivo, and clinical findings, researchers can develop a more comprehensive understanding of the influence of γ-PGA on dermal health and its prospective applications in therapeutic or cosmetic contexts.

## 3. Materials and Methods

### 3.1. Bacterial Strain Isolation and Identification

The bacteria used in this experiment were collected from Gotjawal Wetland in Jeju Island, diluted in a suitable amount of phosphate-buffered solution (pH 7.4), and heated for 5 min at 100 °C. A proportion of these bacteria was cultured on a plate (37 °C, 24 h) with TSA (Merck, Darmstadt, Germany) to isolate the strain. To isolate a pure strain, the morphological differences in the cultured microbes were first screened. The initially selected strains were subjected to multiple rounds of subculturing in fresh medium, and the strain that produced the highest amount of the most viscous PGA was isolated in pure culture. Next, for use in our study, bacteria were cultured in TSA medium, followed by the addition of 30% glycerol and storage at −70 °C.

We performed 16S rRNA sequencing to identify the isolated strains. Following inoculation into tryptic soy broth (Merck, Darmstadt, Germany), the bacteria were incubated for 16 h at 37 °C, centrifuged at 180 rpm, and collected; thereafter, DNA was extracted using a DNeasy Blood & Tissue Kit (QIAGEN, Hilden, Germany). The 16S rRNA gene was synthesized using the universal primers 27F and 1492R. The gene fragment was amplified using PCR, and the amplified PCR product was purified using a QIAquick PCR Purification Kit (QIAGEN, Hilden, Germany). The purified product was sent to SolGent (Daejeon Main Branch, Daejeon, Republic of Korea) for sequencing. In addition, bacteria were identified using an API 50CH Kit (Biomerieux, Marcy-l’Étoile, France) in accordance with Bergey’s Manual of Systematic Bacteriology [41].

### 3.2. Microbial Culture and Reagent Preparation

Tryptic soy broth (Merck, Darmstadt, Germany) was used as the seed culture medium. Considering the data on PGA fermentation [42,43,44,45], a custom medium (10% glutamic acid, 4% glucose, 1.6% citric acid, 1% NH_4_Cl, 0.2% Na_2_HPO_4_, 0.1% KH_2_PO_4_, 0.2% K_2_HPO_4_, 0.005% FeSO_4_, 0.05% MgSO_4_, 0.015% MnSO_4_, 0.015% CaCl_2_, and 0.005% ZnCl_2_) was prepared and used as the main culture medium.

For pre-culture, 2 mL of frozen bacteria was inoculated in a 250 mL Erlenmeyer flask containing 100 mL of tryptic soy broth and incubated aerobically for 24 h at 35 °C and 120 rpm in a shaking incubator. Before the end of the culture period, the culture medium was heated for 10 min at 60 °C to generate a spore suspension that maintained a consistent microbial concentration (2 × 10^8^ CFU/mL) and conditions upon inoculation; the spore suspension was subsequently inoculated into the main culture medium. After inoculating 1.0% (*v*/*v*) of the pre-culture medium in a 250 mL Erlenmeyer flask containing 100 mL of the main culture medium, the bacteria were incubated aerobically for 24 h at 35 °C and 300 rpm in a shaking incubator.

### 3.3. Compound Isolation and Identification

The desired compound was isolated using the methodology described by Goto and Kunioka [46]. Following incubation, the mucous culture medium was diluted 5-fold with distilled water to reduce its viscosity, centrifuged for 120 min at 18,000 rpm, and then passed through a 0.2 μm filter membrane to remove bacteria and collect the supernatant. The pH of the separated supernatant was adjusted to 3 using 6 M HCl, and the supernatant was subjected to acid hydrolysis for 24 h. After acid hydrolysis, the supernatant was mixed with cold ethanol at a ratio of three times the supernatant volume and incubated at 4 °C for 24 h. The obtained compound was filtered through a Whatman filter and dried. The dried compound was dissolved in distilled water at a 1:10 ratio and dialyzed for 4 h using a Slide-A-Lyzer dialysis cassette (10 K MWCO; Thermo Fisher, Waltham, MA, USA) to remove salts. After dialysis, the compound solution was freeze-dried to obtain the samples. Cold ethanol was added to the supernatant at a 4:1 ratio and then left to precipitate for 24 h at 4 °C. After centrifugation for 40 min at 18,000 rpm, the precipitate was collected and freeze-dried to obtain the compound. To obtain a high-purity compound, the freeze-dried compound was completely dissolved in deionized water at a 1:100 ratio, centrifuged for 10 min at 18,000 rpm, and dialyzed for 24 h at 4 °C using a Slide-A-Lyzer dialysis cassette to remove salts, and then the product was freeze-dried to obtain the compound.

To determine the structure of the compound, instrumental analyses such as 1H NMR (D2O) (Avance III, 400 MHz), 13C NMR, and FT-IR (FT/IR-4X, Jasco, Tokyo, Japan) were performed.

The absolute molecular weight of the γ-PGA samples was measured with MALS using a size exclusion column connected to an RI detector. The analysis conditions were as follows: MALS system: Wyatt DAWN Heleos II (18 Angle), Wyatt Optilab T-Rex (RI); software: ASTRA 6; HPLC: Shimadzu; temperature: 25 °C; column: TSK-gel-G4000 SW, Dn/dc (mL/g): 0.15; flow rate: 0.5 mL/min; injection volume: 100 µL; elution solvent: water.

### 3.4. Cell Culture

The human keratinocyte cell line purchased from ATCC (Manassas, VA, USA) was maintained in DMEM:F12 (3:1 *v*/*v*) supplemented with 10% (*v*/*v*) fetal bovine serum and 1% (*w*/*v*) antibiotics at 37 °C in a humidified atmosphere of 95% air/5% CO_2_ (*v*/*v*). The cells were subcultured every 2–3 d and maintained in a culture dish at 37 °C in a 5% CO_2_ incubator.

### 3.5. Cell Viability

We used the MTT assay to assess cell viability [47]. Briefly, keratinocytes were seeded (2 × 10^4^ cells/mL) in 96-well culture plates and incubated for 24 h. The keratinocytes were treated with γ-PGA at various concentrations (15, 30, 62.5, 125, 250, and 500 µg/mL). After incubation for 72 h, the cells were treated with MTT solution. After 4 h, the supernatants were removed, and the insoluble formazan crystals were completely dissolved in dimethyl sulfoxide. The absorbance (Thermo Fisher Scientific, Waltham, MA, USA) was measured at 570 nm.

### 3.6. RT-PCR

Total RNA was extracted using a Total RNA Extraction Kit (Qiagen, Hilden, Germany). Target gene expression was carried out using a One-Step RT-PCR PreMix Kit (Intron Biotechnology, Seongnam-si, Gyeonggi-do, Republic of Korea) with 200 ng of total RNA. The primer sequences used are shown in Table 1. Bands were detected using electrophoresis on 1.2% (*w*/*v*) agarose gels. Band intensities were estimated using Image J 1.47 software (National Institutes of Health, Bethesda, MD, USA) [48,49].

### 3.7. Immunocytofluorescence Analysis

After the cells were fixed in 4% cold PFA, they were washed twice with phosphate-buffered saline (PBS). The cells were then incubated in immunofluorescence staining buffer (PBS, 0.3% Triton X-100, and 5% serum) for 20 min at 21 °C. The cells were incubated with primary antibodies overnight at 4 °C and washed with PBS for 5 min. The cells were incubated with secondary antibodies for 1 h at room temperature and then washed with PBS before mounting with a mounting medium containing 4′,6-diamidino-2-phenylindole.

### 3.8. Reconstructed Skin Model

Full-thickness 3D human skin equivalents were prepared using a previously described method [30]. Briefly, human keratinocytes, human melanocytes, and fibroblasts were cultured in growth media. The dermal equivalent was obtained by producing blocks of fibroblasts with bovine type I collagen and then shrinking them for 5 days at 37 °C. The human keratinocytes and human melanocytes were co-seeded at the top of the contracted dermal equivalent. For monolayer formation, the cultures were immersed in a co-culture growth medium as previously described [30] for 3 days. The culture was then placed in an air–liquid interface and allowed to differentiate into strata for 12 days.

The reconstructed skin tissue (RST) was cultured in six-well plates with coculture growth medium (80% KGM, 1.5 mM CaCl_2_, and 20% MGM) in the presence or absence of γ-PGA on the insert using topical application for 5 days. The RSTs were collected and fixed in 10% neutral formalin solution on day 5. After 24 h, the RSTs were embedded in the optimal cutting temperature (OCT) compound; the frozen OCT blocks were cut into 12 μm sections.

### 3.9. Histological Analysis

For morphological evaluation, 12 μm sections were stained with hematoxylin and eosin through standard methods.

For the immunofluorescence analysis of the OCT sections, after heat-mediated antigen retrieval, the tissue sections were incubated in PBS containing 3% BSA to block non-specific binding. The sections were then incubated with primary antibodies diluted in PBS/BSA 3% overnight at 4 °C. The samples were incubated with secondary AlexaFluor-488- or Alexa-555-conjugated anti-mouse or anti-rabbit antibodies (Abcam, Cambridge, UK) for 1 h at room temperature.

### 3.10. Image Acquisition and Analysis

The sections were mounted using a mounting medium containing 4′,6-diamidino-2 phenylindole and observed under a THUNDER Imager Tissue microscope (Leica Microsystems, Wetzlar, Germany).

Immunostaining was analyzed semi-quantitatively based on the positive area in the photographs using LAS X software, version 3.7.1 (Leica Microsystems). Antibody-positive areas were measured as follows: three different sections from the RSTs (n = 3 RSTs/group) were analyzed, and the percentage of stained area ((positive area/total area) × 100) was calculated. The total area included all layers of the epidermis.

### 3.11. Statistical Analysis

The results are presented as the mean ± standard deviation. The significance of the test substances was confirmed to be within the biological statistical criteria. Statistical significance was set at * *p* < 0.05, ** *p* < 0.01 using Student’s *t*-test on Microsoft Excel 2016.

## 4. Conclusions

This study was conducted to confirm the structure of the mucus substance produced by a strain isolated from Gotjawal Wetland, Jeju Island, and to confirm the skin barrier-strengthening and -moisturizing effects of this substance. The substance secreted by the newly isolated *Bacillus* sp. was confirmed to be γ-PGA.

The formation of a differentiated epidermis in the skin plays an important role in the development of a normal skin barrier. After treating keratinocytes with γ-PGA, it was confirmed that the expression of *FLG*, *IVL*, and *LOR* involved in differentiation increased. The expression of SPT, FAS, and HMG-CoA enzymes involved in the production of lipids which form the skin permeability barrier also increased.

To maintain skin hydration, the development of the skin barrier and the smooth maintenance and transport of moisture within the epidermal layer are necessary. Analysis of the effect of γ-PGA on the synthesis of HA, which has an excellent moisture-retention capacity, confirmed that the amount of HA increased through the increase in HAS-1, -2, and-3 enzyme levels. In addition, we confirmed that the expression of the AQP3 gene and protein, which is a moisture transport channel, increased.

Reconstructed skin is composed of a dermal layer with fibroblasts and an epidermal layer with keratinocytes. When reconstructed skin is exposed to air and cultured, keratinocytes differentiate and form the SC. When the reconstructed skin was treated with γ-PGA, the expression of FLG, IVL, AQP3, and CD44 increased, confirming its skin barrier-strengthening and -moisturizing effects and demonstrating its potential as a cosmetic ingredient. Based on these results, we believe that γ-PGA produced by bacterial strains isolated from Gotjawal Wetland has high applicability as a cosmeceutical ingredient to improve the skin barrier function and skin moisture levels.

The authors of future studies can explore the long-term effects of γ-PGA on skin barrier function and its potential application in treating skin conditions such as eczema and psoriasis. Additionally, they could focus on the optimization of the production process for γ-PGA and its potential application in other fields such as cosmetics and pharmaceuticals.

## Figures and Tables

**Table 1:** Sequences of the PCR primers of the genes investigated.

Gene	Primer	Sequence (5′ to 3′)
*FLG*	Sense	AAGCTTCATGGTGATGCGAC
	Antisense	TCAAGCAGAAGAGGAAGGCA
*IVL*	Sense	ACCTAGCGGACCCGAAATAA
	Antisense	TGGAACAGCAGGAAAAGCAC
*LOR*	Sense	CACTGGGGTTGGGAGGTAGT
	Antisense	GCTCTCATGATGCTACCCGA
*SPT*	Sense	CTGCTGAAGTCCTCAAGGAGTA
	Antisense	GGTTCAGCTCATCACTCAGAATC
*HMG-CoA*	Sense	GATCCAGGAGCGAACCAA
	Antisense	GCGAATAGACACACCACGTT
*FAS*	Sense	CCTCACTGCCATCCAGATTG
	Antisense	CTGTTTACATTCCTCCCAGGAC
*HAS-1*	Sense	CCACCCAGTACAGCGTCAAC
	Antisense	CATGGTGCTTCTGTCGCTCT
*HAS-2*	Sense	TTTGTTCAAGTCCCAGCAGC
	Antisense	ATCCTCCTGGGTGGTGTGAT
*HAS-3*	Sense	CCCAGCCAGATTTGTTGATG
	Antisense	AGTGGTCACGGGTTTCTTCC
*GAPDH*	Sense	CAAAGTTGTCATGGATGACC
	Antisense	CCATGGAGAAGGCTGGGG

## Data Availability

Data can be obtained by contacting the corresponding author.
